# Applications of Bioinformatics and Systems Biology in Precision Medicine and Immunooncology

**DOI:** 10.1155/2018/1427978

**Published:** 2018-09-30

**Authors:** Yudong Cai, Tao Huang, Jialiang Yang

**Affiliations:** ^1^School of Life Sciences, Shanghai University, Shanghai 200444, China; ^2^Shanghai Institutes for Biological Sciences, Chinese Academy of Sciences, Shanghai 200031, China; ^3^Department of Genetics and Genomic Sciences, Icahn School of Medicine at Mount Sinai, New York City, NY 10029, USA

## 1. Introduction

Next-Generation Sequencing (NGS) technology, often seen as the foundation of precision medicine, has been successfully applied in oncology diagnostics and immunotherapy. With advances in gene diagnostics and immunotherapy, there may be a chance to control the development of cancers and alleviate the suffering of patients undergoing chemotherapy. To promote the translation of precision medicine from bench to beside and from application of genetic testing to personalized medicine, new analysis methods for NGS and genetic data need to be developed. For example, the NGS panel is quite different from whole genome sequencing (WGS), focusing on fewer genes or regions but requiring greater precision and efficiency. For complex diseases, such as cancers, the driver genes are usually a cluster of genes in a regulatory network. Graph theories, such as shortest path analysis and random walk algorithms, will help dissect genomewide interactions into key modules or paths whose dysfunction is associated with disease progression.

In this special issue, we have received 32 papers, out of which 19 has been accepted for publication. These papers could be generally divided into 3 categories including (1) computational models in identifying key biomarkers, pathways, and network modules associated with cancers and other diseases, (2) validations of the mechanisms of key biomarkers and their applications in tumor diagnosis and treatment, and (3) other studies in predicting tumor evolution, drug-disease association, disease sequence alignment, and so on.

## 2. Computational Models in Identifying Key Biomarkers

H. Choi and K. J. Na first identified survival-related gene network modules. By selecting representative genes from survival-related modules, they developed a deep learning-based risk stratification model for lung cancer. Their model showed high predictability for prognosis in independent datasets and its predictive value was independent of clinical and pathological features of lung cancer.

J. Zheng et al. proposed a method for constructing the pathway network of gene phenotype. Briefly, it firstly builds a biological pathway network, and then the GeneRank algorithm was used to select disease-associated pathways. The results by gene expression data of breast cancer show that the method proposes an effective way to identify reliable disease-associated pathways.

X. Li et al. presented a new computational method based on the SimRank and density-based clustering recommender model for miRNA-disease association's prediction (SRMDAP). The AUC of 0.8838 based on leave-one-out cross validation and case studies suggested the excellent performance of the SRMDAP in predicting miRNA-disease associations. SRMDAP could also predict diseases without any related miRNAs and miRNAs without any related diseases.

S. Zhu et al. provided a sample-specific method that constructs an index with individual-specific dynamical network biomarkers (DNB), which are defined as early warning index (EWI) for detecting predisease state of individual sample. Based on microarray data of influenza A disease, 144 genes are selected as DNB and the 7th time period is defined as predisease state.

B. Xie et al. first identified differentially expressed genes between patients with poor chemotherapy reaction and patients with good chemotherapy reaction, followed by GO and KEGG pathway analyses, PPI network analysis, survival analysis of hub genes, and miRNA target prediction. This study provides a better understanding about gene expression in drug resistance samples, which is potentially useful for the treatment of osteosarcoma.

## 3. Validations of Key Biomarkers and Their Applications in Tumor Diagnosis and Treatment

H. Meng et al. investigated the prevalence and prognostic and clinicopathologic features of human papillomavirus-related oropharyngeal cancer in northeast China and elucidated the involvement of p16 in the tumorigenesis and progression of oropharyngeal squamous cell carcinoma (OPSCC) from 1470 OPSCC patients collected from 2000 to 2016. They also demonstrated that p16 expression is significantly associated with early stage primary OPSCCs and the patients with p16 expression tend to show better survival following surgery and radiotherapy.

X. Wu et al. investigated the clinicopathologic and prognostic significance as well as the potential role of* HMGB1* in the development and progression of lung cancer. Their results suggest that* HMGB1* expression is significantly associated with lung cancer progression and might be a potential prognosis and therapeutic marker for lung cancer.

H.-B. Xing et al. investigated whether the knockdown of* IL-6* enhances the gemcitabine sensitivity of PANC-1 cells. In their experiments, the knockdown of* IL-6* induced apoptosis and reduced cell proliferation and tumorigenicity and remarkably promoted the antitumor effect of gemcitabine. The results suggest that combining shRNA targeting* IL-6* and gemcitabine is a potential clinical approach for pancreatic cancer therapy.

R. Liu et al. detected RAS mutation, immunoscore, and PD-L1 expression in 60 Chinese metastatic colorectal cancer (mCRC) patients and suggested PD-L1 expression and RAS status to be prognostic indicators for mCRC patients with palliative operation.

X. Yu et al. investigated the mechanism of AEG-1 for regulating metastasis in hypoxia-induced ovarian carcinoma. By a statistical method comparing the protein amounts of AEG-1, HIF-1*α*, and VEGF in ovarian cancer tissue specimens, they hypothesized that AEG-1 associates with hypoxia in ovarian cancer by regulating the HIF-1-alpha/NF-kappa-B/ VEGF pathway.

Y. Chen et al. examined PD-L1 and PD-1 expression in thymic epithelial tumor (TET) tissues from patients. They found that PD-L1 expression levels were correlated with disease progression. They also showed that PD-L1 mRNA levels were also correlated with its immunohistochemistry staining levels and thus can be used as alternative method to detect PD-L1 levels in TETs.

X. Wu et al. also revealed that glycyrrhizin reduces the activity of JAK/STAT signaling pathway, which regulates the expression of* HMGB1*. This is a potential mechanism by which glycyrrhizin can inhibit the progression of lung cancer.

Z. Jiang et al. investigated the prognostic value of liver function in colorectal liver metastases patients. They showed that biochemical analyses of liver function tests at the initial diagnosis of colorectal liver metastases enable the stratification of patients into low- and high-risk groups, which may help clinicians to determine promising treatment strategies.

## 4. Other Studies in Predicting Tumor Evolution, Drug-Disease Association, and Disease Sequence Alignment

Y. Liang et al. proposed an improved binary differential evolution algorithm, BDEP, to infer tumor phylogenetic tree based on fluorescent in situ hybridization (FISH) platform. The constructed phylogenetic trees have great performance in characterizing tumor development process, which outperforms other similar algorithms.

H. Cui et al. developed a method called PEDD to predict the effect of drugs on diseases by using gene expression profiles from both disease-related tissues and cell lines treated with drugs. The authors also implemented the method with an interactive web tool and applied the method to real microarray and RNA-seq datasets.

Finally, M. Li et al. proposed a novel protein sequence alignment method, which outperforms a few current alignment methods in accuracy. Y. Luo et al. developed a computational method to investigate key GO terms and KEGG pathways associated with copy number variations (CNVs). Y. Fang et al. explored the genetic polymorphism of* Aedes albopictus* and its association with diseases like dengue. And L. Wang et al. studied the roles of swine as a mixtures for influenza viruses.

In summary, we expect that this special issue updates novel NGS-based computational methods in predicting key genes, pathways, and network modules associated with diseases like cancers and experimental validations of their mechanisms, as well as their applications in disease diagnosis and treatment. The studies will serve as a bridge to connect computational models in mining clinical NGS data and translation of the findings into personalized therapies for diseases.

